# Global SUMOylation is a molecular mechanism underlying hypothermia-induced ischemic tolerance

**DOI:** 10.3389/fncel.2014.00416

**Published:** 2014-12-04

**Authors:** Yang-ja Lee, Yongshan Mou, Dace Klimanis, Joshua D. Bernstock, John M. Hallenbeck

**Affiliations:** Stroke Branch, National Institute of Neurological Disorders and Stroke, National Institutes of HealthBethesda, MD, USA

**Keywords:** Hypothermia, SUMOylation, pMCAO, Neuroprotection, Ubc9

## Abstract

The molecular mechanisms underlying hypothermic neuroprotection have yet to be fully elucidated. Herein we demonstrate that global SUMOylation, a form of post-translational modification with the Small Ubiquitin-like MOdifer, participates in the multimodal molecular induction of hypothermia-induced ischemic tolerance. Mild (32°C) to moderate (28°C) hypothermic treatment(s) during OGD (oxygen-glucose-deprivation) or ROG (restoration of oxygen/glucose) increased global SUMO-conjugation levels and protected cells (both SHSY5Y and E18 rat cortical neurons) from OGD and ROG-induced cell death. Hypothermic exposure either before or after permanent middle cerebral artery occlusion (pMCAO) surgery in wild type mice increased global SUMO-conjugation levels in the brain and in so doing protected these animals from pMCAO-induced ischemic damage. Of note, hypothermic exposure did not provide an additional increase in protection from pMCAO-induced ischemic brain damage in Ubc9 transgenic (Ubc9 Tg) mice, which overexpress the sole E2 SUMO conjugating enzyme and thereby display elevated basal levels of global SUMOylation under normothermic conditions. Such evidence suggests that increases in global SUMOylation are critical and may account for a substantial part of the observed increase in cellular tolerance to brain ischemia caused via hypothermia.

## Introduction

Stroke is the fourth leading cause of death and a leading cause of long-term disability in adults (Roger et al., 2012). While a myriad of cellular and molecular brain injury mechanisms have been reported in preclinical stroke models (reviewed in Dirnagl et al., 1999; Mergenthaler et al., 2004; Broussalis et al., 2012a), the individual targeting of these mechanisms has thus far not proven effective (reviewed in O’Collins et al., 2006, 2009; Broussalis et al., 2012b). In the wake of such failures ischemic brain damage has gradually become viewed as a highly complex, multifactorial process that involves the interplay of many non-dominant effectors (Hallenbeck and Frerichs, 1993; Hallenbeck, 2002; Iadecola and Anrather, 2011).

In contrast to the aforementioned failures, hypothermia has been recognized as perhaps the most robust brain cytoprotectant studied in the laboratory to date (reviewed in González-Ibarra et al., 2011; Yenari and Han, 2012). The neuroprotective effects conferred by hypothermia are multifactorial. As such, hypothermia has been shown to alter a number of the pathologic molecular mechanisms induced by cerebral ischemia. Such changes include reductions in metabolic and enzymatic activity, glutamate release and re-uptake, inflammation, the production of reactive oxygen species, and expression of various genes (reviewed in González-Ibarra et al., 2011; Yenari and Han, 2012). Preliminary clinical studies utilizing mild to moderate hypothermia as a treatment for acute ischemic stroke are ongoing and results obtained thus far have been encouraging (Macleod et al., 2010; van der Worp et al., 2010; Abdullah and Husin, 2011; Kollmar et al., 2012). Despite such work and the reproducible demonstrations of hypothermic neuroprotection by a number of laboratories, the cellular and molecular mechanisms underlying brain cytoprotection induced via exposure to hypothermia are not fully understood.

One candidate capable of coordinating the multimodal molecular mechanisms that underlie the cytoprotection induced in the brain as a consequence of cooling is that of global SUMOylation. Post-translational modification by this ubiquitin-like modifier (ULM) appears to have multifunctional effects and operates in states of tolerance to preserve homeostasis under stress (Tempé et al., 2008). We have previously reported on the massive increases in SUMOylation that occur in 13-lined ground squirrels during hibernation torpor, a period in which their body temperature declines to 5°C (deep hypothermic condition) (Lee et al., 2007). We hypothesized such massive increases in SUMO-conjugation were involved in the profound natural tolerance to the reductions in brain blood flow and oxygen delivery displayed by these hibernating animals. Our work and that of others have shown that global SUMOylation is indeed involved in ischemic tolerance in both *in vitro* cell culture systems (Lee et al., 2007, 2009; Datwyler et al., 2011; Cimarosti et al., 2012) and animal models (Cimarosti et al., 2008; Yang et al., 2008; Lee et al., 2011).

SUMO, like ubiquitin, is synthesized as an inactive precursor and subsequently processed by SUMO-specific proteases to yield the mature di-glycine C-terminus. A single heterodimeric E1 activating enzyme (SAE1/SAE2) initiates conjugation by adenylating SUMO. This is followed by the formation of a covalent thioester E1-SUMO intermediate. SUMO is then transferred to the catalytic cysteine of the sole E2 conjugase, Ubc9 (Ubiquitin conjugase 9), which alone or in concert with an E3 ligase catalyzes the formation of an isopeptide linkage between the C-terminal glycine residue of SUMO and the epsilon-amino group of the substrate’s lysine residue. The conjugation actions of Ubc9 are opposed by isopeptidases, which catalyze de-SUMOylation thereby modulating steady state levels of SUMO-conjugates (reviewed in Müller et al., 2001; Hay, 2005). There are three systemically distributed SUMO paralogs in mammals: the sequences of SUMO-2 and SUMO-3 are 96% identical and are therefore difficult to distinguish. In contrast, SUMO-1 is only 45% identical with to the other two SUMO paralogs and has a distinct immunoreactivity (Tatham et al., 2001).

Of note, we have generated Ubc9 transgenic (Ubc9 Tg) mice, which displayed increased levels of SUMO-conjugation and an improved tolerance to permanent middle cerebral artery occlusion (pMCAO)-induced brain damage (Lee et al., 2011). Using these Ubc9 Tg mice, the human neuroblastoma cell line SHSY5Y and E18 derived rat cortical neurons, we present evidence that strongly links hypothermia-induced ischemic resilience to corresponding levels of global SUMOylation.

## Materials and methods

### SHSY5Y cell culture and isolation of rat cortical neurons/culture

The human neuroblastoma cell line SHSY5Y (American Type Culture Collection, Manassas, VA, USA) was cultured in Dulbecco’s modified Eagle’s medium (DMEM) supplemented with 10% heat-inactivated fetal bovine serum, 100 U/ml penicillin, and 100 mg/ml streptomycin (DMEM complete) in 5% CO_2_ at 37°C. Embryos (day 18) of Sprague-Dawley rats were used to prepare cortical neuronal cultures. Cortices were dissected from the embryos, dissociated with papain (Worthington Biochemicals, Lakewood, NJ, USA), and plated out at 1,000,000 cells per well on poly-L-lysine-coated 6 well plates in Neurobasal-A/B27 media (Lee et al., 2009). Cells were used after 7 days in culture.

### Oxygen and glucose deprivation (OGD), restoration of oxygen-glucose (ROG), hypothermia, and the assessment of cell death

Oxygen-glucose-deprivation (OGD) using SHSY5Y cells and cortical neuronal cultures was performed as previously described (Lee et al., 2007, 2009). OGD was carried out either at 37°C (normothermia) or 32°C (hypothermia). After a 12 h (SHSY5Y) or 3–9 h (cortical neurons) incubation, plates were taken out of the OGD chamber, OGD media was replaced with complete media which contained glucose (DMEM for SHSY5Y and Neurobasal-A/B27 for cortical neurons), and incubated for an additional 16 h at 37°C (normothermia), 32°C or 28°C (hypothermia) for restoration of oxygen/glucose (ROG).

Cell death as induced by OGD alone or after ROG was assessed by nuclear staining with Hoechst 33342 and propidium iodide via fluorescence-activated cell sorting (FACS) analysis as described previously (Lee et al., 2007), or followed by counting directly under the fluorescent microscope (Lee et al., 2009).

### Animal experiments

The National Institute of Neurological Disorders and Stroke Animal Care and Use Committee approved all experiments under the protocol #1268-12 entitled “Use of Ubc9 Tg mice for pre-clinical study of stroke.” Ubc9 Tg mice were generated as previously described (Lee et al., 2011). An equal number of male and female adult mice (18–20 weeks old, weighing between 20–30 g) were used throughout the course of all experiments. Of note, within this aged cohort males typically weighed more than females, but brain sizes amongst males and females were comparable and no significant gender differences in susceptibility to pMCAO-induced brain damage were noted in the course of our studies. Animal hypothermia was induced by using the water immersion method described previously (Mou et al., 2013). Using this method, body temperature was tightly controlled and we noted that brain temperature (BT) was consistently recorded to be 0.3–0.5°C higher than the rectal temperature recorded during hypothermic treatment. We therefore used rectal temperature as a surrogate capable of monitoring BT. Animals were eliminated from the study if any of the follow conditions were met: (i) hemorrhage from the MCA; (ii) injury to another branch of MCA; (iii) extreme cerebral blood flow change (i.e., <70% and >90%); and (iv) inconsistent body temperature control (surgery < 36.5°C and >37.5°C) + (hypothermia < 0.2°C and >0.2°C of intended target temperature). The animals used in these experiments were randomized and the surgeon was blinded as to the genetic backgrounds of the animals. The evaluation of infarct size was conducted in manner that blinded one to both the experimental conditions and animal’s genetic background.

Oxygen saturation, heart and respiratory rates were monitored using an Oximeter (STARR Life Sciences Corporation). Focal cerebral ischemia was induced via pMCAO in mice as previously described (Lee et al., 2011). Briefly, a 1.5~2.0 cm incision was made between the left eye and ear. A craniectomy was then performed at the skull base (~2 mm in diameter) to expose, cauterize and transect the left MCA and it’s anterior branch (if available) at the level of the olfactory tract. Of note, 24 h of a focal pMCAO procedure will normally cause an infarct that occupies about one-third of the ipsilateral hemisphere. As such, at 24 h after surgery (unless otherwise described), each animal was anesthetized with isoflurane and decapitated. Brains were promptly removed and snap frozen for the assessment of brain damage and for Western blot analysis of global SUMO-conjugation.

### Western blot analysis

The lysates from SHSY5Y cells or cortical neurons (Lee et al., 2007, 2009) and brain extracts (Lee et al., 2007, 2011) were prepared as previously described. Rabbit polyclonal anti-SUMO-1 and anti-SUMO-2/3 antibodies (both developed in-house) and anti-β-actin antibody (Sigma Chemical) were used. The intensities of bands were analyzed using Image-J (NIH). In order to measure SUMO-conjugation levels, the region corresponding to molecular weights 65 ~300 kDa for cells and ~90 to 250 kDa for mouse brains in each lane were cropped and the total intensity was analyzed (Lee et al., 2007).

### Cresyl violet staining and the assessment of infarction volumes

Frozen brain coronal sections (20 µm) from both WT and Ubc9 Tg mice, which had undergone 24 h of pMCAO were stained with cresyl violet; brain damage was subsequently assessed as previously described (Lee et al., 2011). Briefly, quantification of the infarct area was performed on 20 µm cresyl-violet-stained sections collected at 0.34 mm intervals. The infarct area of each section was measured using Image J. The effect of edema was corrected for using the following equation (Leach et al., 1993): corrected infarct area = infarct area × area of contralateral hemisphere / area of ipsilateral hemisphere. Infarct volume (mm^3^) was calculated for each animal by integrating the corrected infarct area with the distance between sections (0.34 mm) (Arsenijevic et al., 2006).

### Statistics

Variables were analyzed (two group comparison) using a Mann-Whitney U test with a Bonferroni correction. *p* < 0.05 was considered statistically significant. All values are expressed as a mean ± SD (standard deviation) of at least three independent experiments with at least 6 animals contained within each group. Correlations between two variables of interest were obtained using Spearman’s correlation coefficient. *p* < 0.05 was considered to be statistically significant.

## Results

### Hypothermia exposure increases global SUMO-conjugation levels and protects SHSY5Y cells and rat cortical neurons from OGD-induced cell death

**Figure 1:**
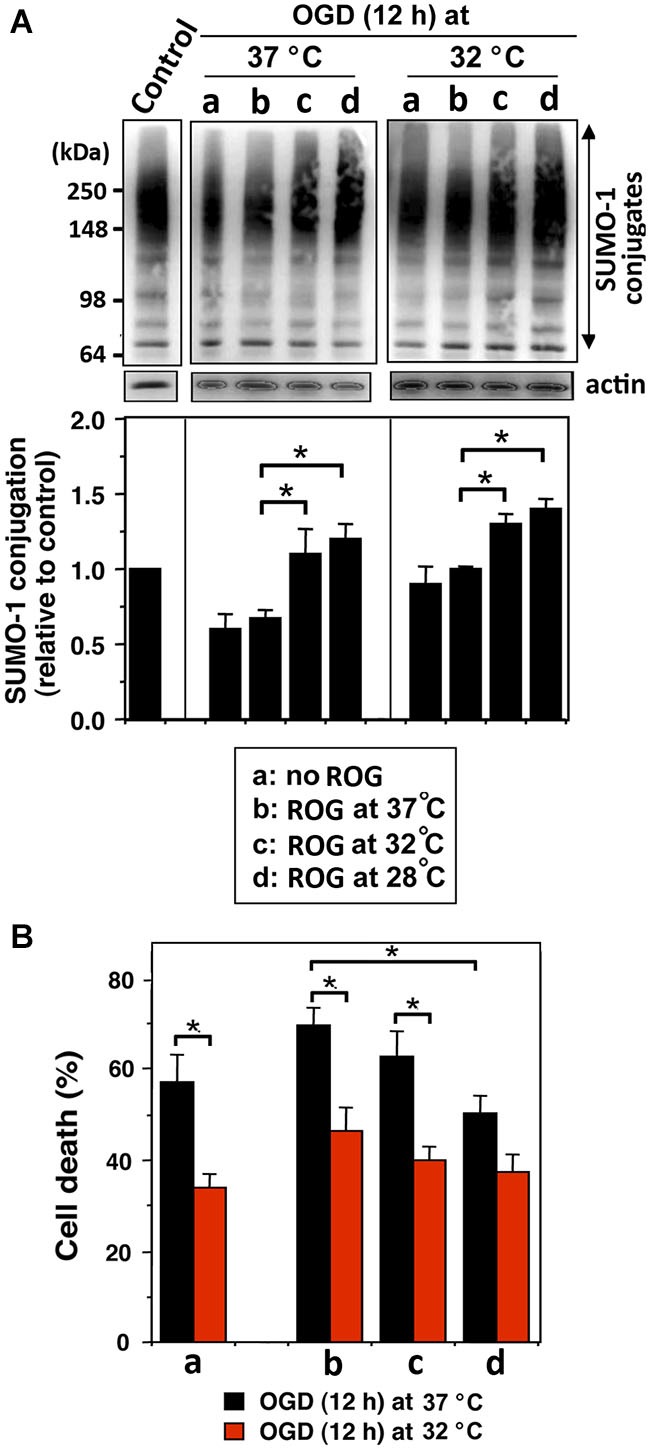
**Hypothermia treatments during OGD and/or ROG increase SUMO-1 conjugation levels and protect cells from OGD or OGD/ROG-induced death in SHSY5Y cells**. SHSY5Y cells were subjected to OGD either at 37°C or 32°C for 12 h (a) followed by ROG at 37°C (b) 32°C (c) or 28°C (d) for 16 h. **(A)** SUMO-1 conjugation levels at each time point. The upper panel is a representative immunoblot and the lower panel presents a quantitation of SUMO-1 conjugation levels. Densities of higher molecular weight conjugates (65 ~ 300 kDa) were measured, normalized to corresponding actin levels and shown as relative to control (neither OGD nor ROG-treated cells). Data are presented as the mean ± SD of three independent experiments. *p < 0.05 compared to normothermia during ROG. **(B)** Cell death was assessed by nuclear staining with Hoechst 33342 and propidium iodide followed by fluorescence activated cell sorting (FACS) analysis after OGD (a) or followed by ROG at 37°C (b), 32°C (c) or 28°C (d). Data are presented as the mean ± SD of three independent experiments. *p < 0.05 compared to the normothermia group during ROG.

**Figure 2:**
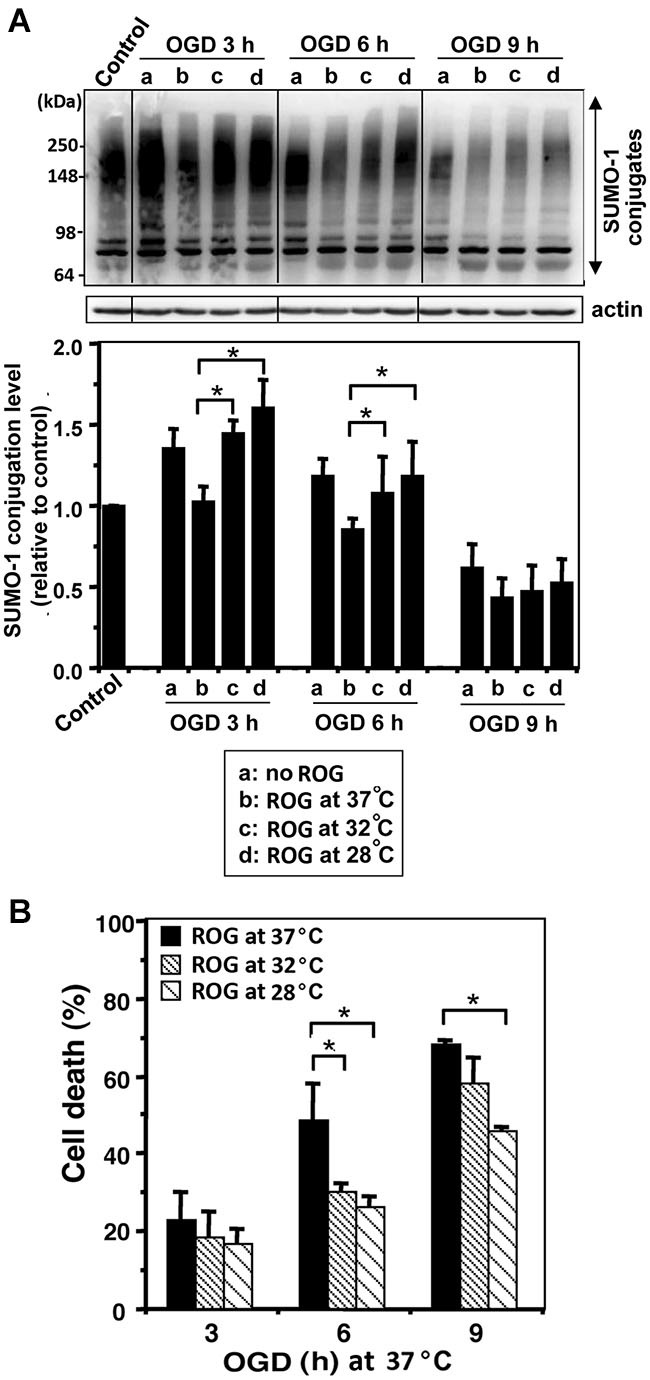
**Hypothermic treatments during ROG increase SUMO-1 conjugation levels and protect cells from OGD/ROG-induced death in rat cortical neurons**. Rat cortical neuronal cultures were subjected to OGD at 37°C for 3, 6 or 9 h followed by ROG at 37°C (b) 32°C (c) or 28°C (d) for 16 h. **(A)** SUMO-1 conjugation levels at each time point: control, OGD alone (a) and OGD/ROG (b: 37°C, c: 32°C, d: 28°C). Representative immunoblots (upper panel) and quantitation of SUMO-1 conjugation levels (lower panel). Densities of high molecular weight conjugates (65 ~ 300 kDa) were measured, normalized to corresponding actin levels and shown as relative to controls that did not receive an OGD or ROG exposure. Data are presented as the mean ± SD of three independent experiments. **(B)** Cell death was assessed by nuclear staining with Hoechst 33342 and propidium iodide followed by counting directly under the fluorescent microscope (≥300 cells) after OGD at 37°C and ROG at 37°C, 32°C, or 28°C. Data are presented as the mean ± SD of three independent experiments. *p < 0.05 compared to normothermic OGD group.

We examined whether mild (32°C) to moderate (28°C) hypothermia, both of which fall within the temperature range used to induce clinical hypothermia, would be capable of increasing SUMO-conjugation levels, and if so, would such an increase be beneficial in an effort to protect cells from ischemic damage. We first examined SHSY5Y cells for SUMO-1 conjugation levels during OGD, or OGD/ROG at 37°C, 32°C, or 28°C. As shown in Figure 1A, 12 h OGD at 37°C decreased SUMO-1 conjugation (40% decrease from control), yet the decrease was much smaller (less than 20%) when cells were subjected to OGD at 32°C. Of note, the levels of global SUMO-conjugation did not change further when ROG was carried out at 37°C, but the conjugation levels significantly increased when ROG was carried out at either 32°C or 28°C. These results clearly indicate that hypothermic treatments can both preserve during OGD and increase during ROG the levels of SUMO-1 conjugation in SHSY5Y cells. As shown in Figure 1B, 12 h of OGD at 37°C resulted in ~60% cell death, but the same length of OGD at 32°C reduced cell death to a mere ~35%. Further, cell death induced by OGD at 37°C continue to increase following ROG at 37°C to 70%, but was significantly reduced (to ~50%) when ROG was carried out at 28°C. As such, in line with previously published reports hypothermic treatment inhibits cell death caused by either OGD alone or OGD/ROG in SHSY5Y cells. Next, we carried out a similar set of experiments utilizing primary cultures of E18 rat cortical neurons. We examined the effects of hypothermic treatment on both global SUMO-1 conjugation levels and cell death during ROG. Primary cortical neurons were subjected to OGD at 37°C for 3, 6, or 9 h, followed by ROG at 37°C, 32°C, or 28°C. As shown in Figure 2A, short durations of OGD increased SUMO-1 conjugation (i.e., preconditioning), but conjugation was drastically decreased by longer/more lethal OGD. Of note, the SUMO-1 conjugation that was increased by shorter periods of OGD gradually decreased during ROG at 37°C. However, SUMO-1 conjugation levels in these cells were restored and/or increased when ROG was carried out under hypothermic conditions (32°C or 28°C). In our experimental setting, 3 h of OGD followed by 16 h of ROG resulted in a modest amount of cell death in cortical neurons, and thus the protective effects of hypothermia were minimal (Figure 2B). 6 h of OGD followed by 16 h ROG at 37°C caused over 50% cell death in cortical neuronal cells, but hypothermia treatment during ROG was capable of significantly decreasing cell death (Figure 2B). 9 h of OGD was very severe for the cortical neurons, yet hypothermic treatment during ROG (especially at 28°C) was capable of inducing significant protection (Figure 2B).

### WT mice exposed to hypothermia before pMCAO surgery showed elevated SUMO-conjugation levels in the brain and were protected from pMCAO-induced brain damage, while Ubc9 Tg mice displayed no additional protection after hypothermic treatment

**Figure 3:**
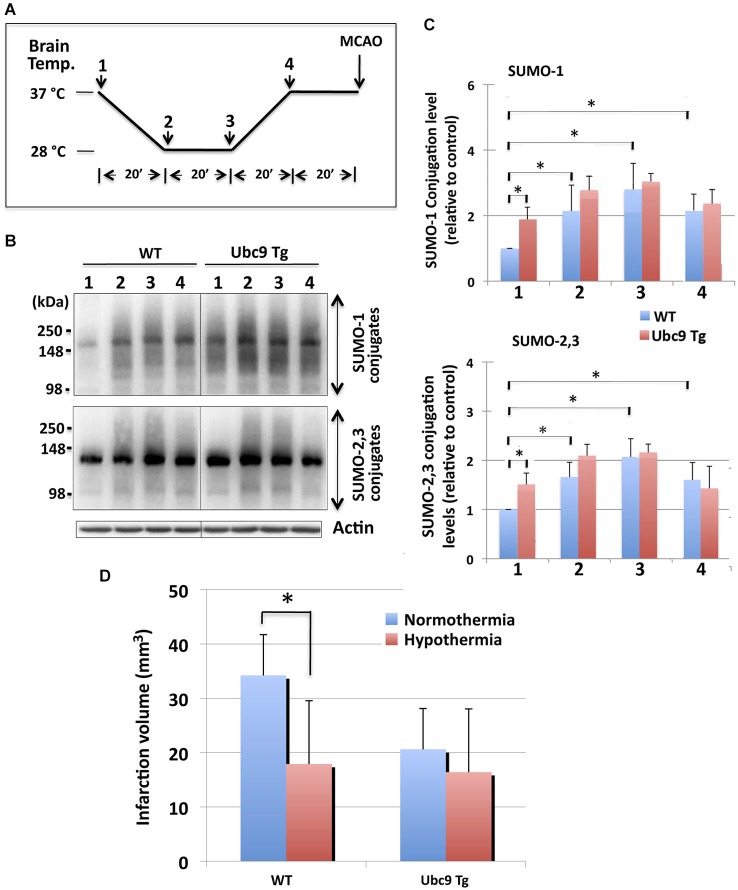
**Effects of hypothermia exposure before pMCAO surgery on brain SUMOylation levels and on subsequent pMCAO-induced ischemic brain injury. (A)** Diagram of serial temperature stages used during hypothermic exposure of mice. Animals (WT and Ubc9 Tg) (1) were cooled to 28°C (BT) over a period of 20 min (2), maintained at a BT of 28°C for 20 min (3), BT was increased to 37°C over a period of 20 min (4), and BT was maintained at 37°C for 20 min before pMCAO surgery. **(B)** Representative immunoblots for SUMO-1 conjugation (upper panel) and SUMO-2/3 conjugation (lower panel) in whole brain extracts. At time/temperature points 1–4, animals were euthanized and brains were promptly removed and frozen for the Western blot analysis of SUMO conjugation. Whole brain extracts were prepared and equal amounts of protein (20 µg) were analyzed for SUMO conjugation. Actin levels were also examined in the same blot. **(C)** Quantitative analyses of SUMO-1 conjugation (upper panel) and SUMO-2/3 conjugation (lower panel) at each time point. Densities of higher molecular weight conjugates (90 ~ 250 kDa) were measured, normalized to the corresponding actin level and shown relative to control (WT, without hypothermia). Blue: WT, and Red: Ubc9Tg mice. Data are presented as the mean ± SD of 6 animals per group (WT and Ubc9Tg) at each time point. **(D)** Hypothermia exposure before pMCAO surgery significantly reduces pMCAO-induced infarction volume in WT mice, but not in Ubc9 Tg mice. WT and Ubc9 Tg mice that had been exposed to hypothermia and their normothermic control animals in which brain temperatures had been maintained at 37°C under anesthesia for the same total duration as the hypothermia experimental groups (80 min) were subjected to pMCAO, and 24 h later the brain infarction volumes were measured. Blue: normothermia, Red: hypothermia. Data are presented as the mean ± SD of 6 WT and 6 Ubc9 Tg animals in each of the two conditions (normothermia and hypothermia). *p < 0.05 compared to normothermia.

We followed the hypothermic preconditioning paradigm that was initially used in rats (Yunoki et al., 2003) for our mouse experiments. In so doing, we compared WT mice with mice that were transgenic for Ubc9, the sole E2 SUMO conjugase. The hypothermic treatment paradigm employed is diagrammed in Figure 3A: BT of WT and Ubc9 Tg animals was cooled to 28°C over a period of 20 min, maintained at 28°C for 20 min, then increased to 37°C over a period of 20 min, and finally maintained at 37°C for 20 min before the pMCAO surgery. The BT of the normothermia group was kept at 37°C for 80 min under anesthesia before the pMCAO surgery. SUMOylation levels throughout the entire brain were analyzed at each time point during the hypothermic treatment. As shown in Figures 3B,C, SUMO-1 and SUMO-2/3 conjugation levels in the brain were increased when BT was maintained at 28°C (stages 2–3). Of note some of the increases persisted even when the BT was returned to 37°C (stage 4) in WT animals. The Ubc9 Tg mice demonstrated trends similar to WT, yet the overall conjugation levels were predominantly higher than WT. Interestingly, SUMO-1 (and SUMO-2/3) conjugation levels at stage 4, just before pMCAO surgery, were essentially identical for WT and Ubc9Tg mice. When hypothermia-pretreated WT mice were subjected to pMCAO for 24 h, brain damage (infarction volume) was significantly less (~50% reduction) as compared to normothermic WT animals (Figure 3D). As reported in our previous paper (Lee et al., 2011), Ubc9 Tg mice were more resistant to pMCAO even under normothermic condition, and no further reduction in brain damage was achieved via hypothermic treatment (Figure 3D).

### Hypothermic treatment of WT mice after pMCAO-surgery elevated SUMO-conjugation levels in the brain and decreased pMCAO-induced brain damage, while Ubc9 Tg Mice that received the same treatment paradigm displayed no further protection

**Figure 4:**
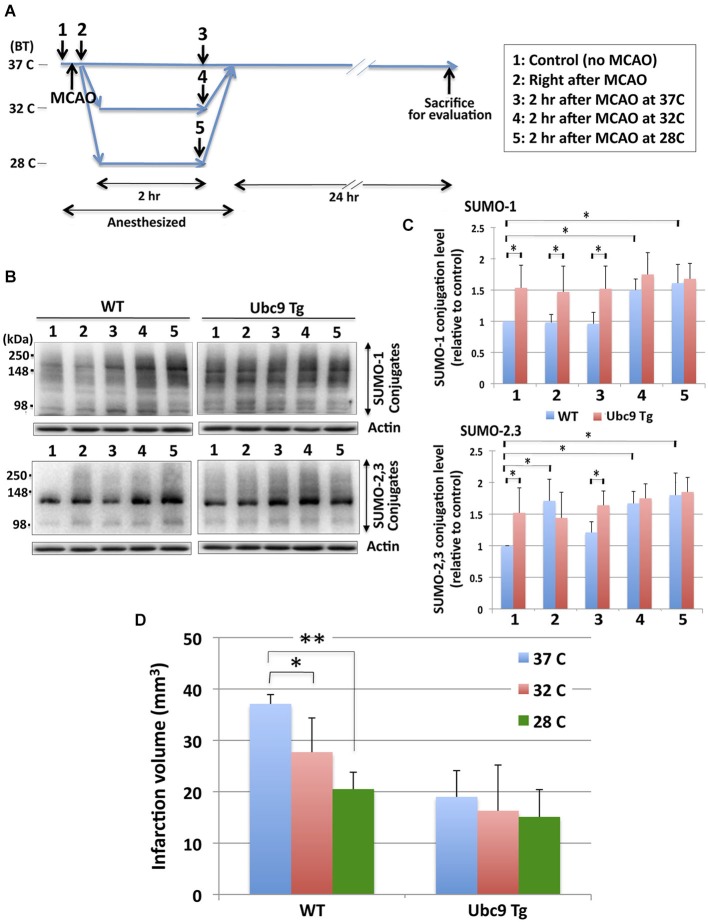
**Effects of hypothermia exposure after pMCAO surgery on brain SUMOylation levels and pMCAO-induced brain injury. (A)** Diagram of hypothermia treatment protocol used in the mice. Animals (WT and Ubc9 Tg) (1) were subjected to pMCAO surgery (2). After surgery one group of WT and Ubc9 Tg animals had BTs maintained at 37°C for 2 h (3) another group at 32°C for 2 h (4), and a third group at 28°C for 2 h (5) under anesthesia. All animals recovered from anesthesia and were housed at room temperature with BTs equal to ~37°C for 24 h before evaluating brain damage. **(B)** Representative immunoblots for SUMO-1 (upper panel) and SUMO-2/3 (lower panel) conjugation in the whole brain extracts from WT and Ubc9 Tg mice. At each time point (1–5), 6 animals in each group were euthanized, brains were promptly removed and frozen for Western blot analysis. Whole brain extracts were prepared and equal amounts of protein (20 µg) were analyzed for SUMO conjugation. Actin levels were also examined in the same blot as loading controls. **(C)** Quantitation of SUMO-1 (upper panel) and SUMO-2/3 (lower panel) conjugation levels at each time point in WT and Ubc9 Tg. Densities of higher molecular weight conjugates (90 ~ 250 kDa) were measured, normalized with corresponding actin levels and shown relative to controls (WT with no pMCAO). Blue: WT, and Red: Ubc9 Tg. Data are presented as the mean ± SD of 6 animals from the WT and from the Ubc9 Tg groups at each time point. **(D)** Hypothermia exposure after pMCAO surgery significantly reduces pMCAO-induced infarction volume in WT mice, but not in Ubc9 Tg mice. Animals (WT and Ubc9 Tg) (18 animals from each group) that had received pMCAO surgery were divided into 3 temperature groups each with 6 WT and 6 Ubc9 Tg: one temperature group’s BTs were maintained at 37°C for 2 h; another at 32°C for 2 h; and another at 28°C for 2 h under anesthesia. All animals recovered from anesthesia and were housed at room temperature for 24 h and evaluated for brain infarct volumes. Blue: 37°C, red: 32°C, green: 28°C. Data are presented as the mean ± SD of six of each animals (WT and Ubc9 Tg) group in each condition. *p < 0.05; **p < 0.01.

In order to examine the effects of hypothermia on the pathologic outcomes induced by the pMCAO surgery, animals (WT and Ubc9 Tg) were subjected to surgery first after which their BTs were maintained either at 37°C, 32°C or 28°C for 2 h. Twenty four hours later brains were harvested and ischemic damage assessed. The treatment paradigm we employed is diagrammed in Figure 4A. First, we examined the SUMO conjugation levels in WT and Ubc9Tg brains at each time point (1–5). As shown in Figures 4B,C, SUMO-conjugation levels (both SUMO-1 and SUMO-2/3) in WT mice were significantly lower than those in Ubc9Tg mice prior to pMCAO (stage 1, normothermia). SUMO-2/3 conjugation, but not SUMO-1 conjugation in WT mice, was increased by the pMCAO surgery (stage 2). SUMO-1 and SUMO-2/3 conjugation levels were slightly decreased or unchanged during the recovery stage at 37°C (stage 3) in WT mice. Critically, when BTs were kept at either 32°C (stage 4) or 28°C (stage 5) after pMCAO surgery, SUMO conjugation levels in WT mice were increased (stage 3≪ stage 4 < stage 5). SUMO conjugation levels in Ubc9 Tg were generally higher than WT (except SUMO-2/3 at stage 2) and were essentially unchanged by hypothermic treatment. Of note, under hypothermia, especially at 28°C, SUMOylation levels in both the WT and Ubc9 Tg mice were effectively identical (Figures 4B,C). Next, we examined the effect of hypothermia after pMCAO surgery on ischemic brain damage. As shown in Figure 4D, hypothermic treatment (particularly at 28°C) for 2 h after the surgery significantly reduced brain damage in WT mice after pMCAO. In accordance with our previous work Ubc9 Tg mice displayed much less ischemic brain damage in the normothermic group, yet no gains in protection were evident in the hypothermic groups (Figure 4D). We did notice that brain infarction volumes in the 32°C treated animal groups (both WT and Ubc9 Tg) varied greatly. As such, we analyzed the correlation of infarction volumes and SUMOylation levels among these animals (6 WT, 6 Ubc9 Tg), and found that infarction volumes did display a significant inverse linear correlation with that of SUMO-1 conjugation levels (Spearman = −0.60839, *P* = 0.0358), and an inverse linear correlation trend for SUMO-2/3 conjugation levels (Spearman = −0.56643, *P* = 0.0548). These results provide evidence that hypothermia exposure increases tolerance to brain ischemia in a manner that is in direct proportion to its capacity to increase in global SUMOylation.

## Discussion

The cytoprotective effects induced via elevated levels of SUMO-conjugation have been reported in cell lines (Lee et al., 2007, 2009), primary neuronal cultures (Lee et al., 2009; Datwyler et al., 2011; Cimarosti et al., 2012) and in the brains of both rats (Cimarosti et al., 2008; Yang et al., 2008) and mice (Lee et al., 2011). Increases in SUMOylation via hypothermic treatments have been identified in hibernating ground squirrels (Lee et al., 2007), and were further noted in cell lines (Lee et al., 2007), primary neuronal cultures (Loftus et al., 2009; Wang et al., 2012) and rats (Yang et al., 2009; Wang et al., 2012). Herein, we have shown that global SUMOylation is increased and sustained by both mild and moderate hypothermia during OGD and ROG in both SHSY5Y cells (Figure 1A) and rat cortical neurons (Figure 2A). It is prudent to note that in those cells in which SUMOylation was augmented by hypothermia, a concordant increase in tolerance to OGD and OGD/ROG-induced cell death was exhibited (Figures 1B, 2B). It is therefore reasonable to deduce from both our work and that of others that global SUMOylation may be one of the molecular mechanisms underlying hypothermia-induced ischemic neuroprotection.

To further explore this putative mechanism we used transgenic animals whose phenotype involves constitutively elevated levels of global SUMOylation. Our Ubc9 Tg mice have been previously phenotyped and the neuroprotection (post-focal brain ischemia) afforded via the upregulation of SUMOylation in these animals characterized (Lee et al., 2011). A myriad of different experimental animal models for therapeutic hypothermia have been reported (reviewed in Krieger and Yenari, 2004; Nagel et al., 2008): each of which utilizes different depths, durations, and times of onset for hypothermia. Further, different animals and models of stroke induction have been employed (Evdokimov et al., 2008; Zhang et al., 2008). We performed two sets of experiments: one using hypothermia as a preconditioning stress (i.e., hypothermic treatment before an ischemic challenge), and the other using hypothermia as a post-conditioning stress (i.e., as a therapeutic intervention after an ischemic challenge). In so doing, we followed the hypothermic preconditioning paradigm put forth by K.S. Lee’s group (Nishio et al., 2000; Yunoki et al., 2002, 2003) for our mouse experiments. By keeping the BT of WT mice at 28°C for 20 min before a pMCAO challenge (Figure 3A), SUMOylation levels in the brain were shown to be increased (Figures 3B,C) while brain damage was reduced significantly (Figure 3D). Ubc9 Tg mice display baseline global SUMOylation levels higher than those of WT and as such displayed a greater tolerance to pMCAO-induced ischemic brain damage in the normothermic state than WT mice (Lee et al., 2011). However, the Ubc9 Tg mice exhibited no additional benefits from hypothermic preconditioning (Figure 3D) which was in stark contrast to the WT mice. When used in the post-occlusion period, hypothermia also showed significant protection from pMCAO-induced brain damage in WT animals, but not in Ubc9 Tg mice (Figure 4). Again, it is prudent to note that both experimental paradigms could only increase tolerance to brain ischemia if that exposure resulted in a corresponding increase in global SUMOylation. These results strongly suggest that global SUMOylation is an important mechanism in the induction of ischemic tolerance via exposure to hypothermia.

Throughout the course of this study our Ubc9 Tg mice demonstrated no additional gain in ischemic cytoprotection from hypothermic treatment, which was in direct contrast to what we witnessed in our WT controls. If SUMOylation is indeed a critical component of the myriad of protective molecular responses induced by hypothermia, Ubc9 Tg mice (which have constitutively elevated levels of global SUMOylation even under normothermic conditions) would be expected to fail in displaying an increase in brain cytoprotection resultant from hypothermic exposure to the same degree as WT mice. This outcome is in line with our results and provides a direct link between levels of SUMOylation and the litany of protective molecular mechanisms underlying hypothermia-induced cytoprotection post-ischemia. Unfortunately, SUMO-1,2,3 knock-out animals are not yet available with which to investigate whether SUMO-conjugation is an underlying molecular mechanism in hypothermia-induce ischemic tolerance through a series of loss of function experiments. While SUMO-1 knock-out mice have been reported, the majority of SUMO-1 functions were compensated for *in vivo* by SUMO-2 and SUMO-3 (Evdokimov et al., 2008; Zhang et al., 2008). Similarly, when all three SUMO isoforms are prevented from functioning via the overexpression of a dominant negative Ubc9 mutant or by knocking Ubc9 down *in vitro* cells are not capable of surviving (Hayashi et al., 2002; Nacerddine et al., 2005; Lee et al., 2007).

Among the many experimental stroke therapies hypothermia remains one of the most effective and promising neuroprotectants studied to date. However, several obstacles continue to impede its clinical translation. As such, pharmacologically induced hypothermia has been proposed as a very attractive alternative (Katz et al., 2004; Yenari et al., 2008; Choi et al., 2012). If one considers that global SUMOylation may in fact be one of the critical underlying mechanisms of hypothermia-induced cytoprotection, the clinical utility in seeking ways to induce an increase in global SUMOylation pharmacologically becomes readily apparent. Accordingly, we are in the process of identifying and aggressively pursing small molecules that increase global SUMOylation via the high throughput screening of compound libraries in an effort to advance neuroprotection and bring effective molecular therapies to bear.

## Conflict of interest statement

The authors declare that the research was conducted in the absence of any commercial or financial relationships that could be construed as a potential conflict of interest.

## Abbreviations

SUMO: small ubiquitin-related modifier
OGD: oxygen-glucose deprivation
ROG: recovery from oxygen/glucose deprivation
Ubc9: ubiquitin conjugase 9
Tg: transgenic
pMCAO: permanent middle cerebral artery occlusion.
